# ERGA-BGE genome of *Dendarus foraminosus*: an IUCN Least Concern darkling beetle endemic to Crete (Greece)

**DOI:** 10.12688/openreseurope.20489.1

**Published:** 2025-06-30

**Authors:** Giannis Bolanakis, Danae Karakasi, Apostolos Trichas, Astrid Böhne, Rita Monteiro, Rosa Fernández, Nuria Escudero, Eleftherios Bitzilekis, Manos Stratakis, Petros Lymberakis, Nikolaos Poulakakis, Genoscope Sequencing Team, Manon Angel, Jean-Marc Barbance, Julie Batisse, Odette Beluche, Laurie Bertrand, Elodie Brun, Maria Dubois, Corinne Dumont, Barbara Estrada, Thomas Guerin, Zineb El Hajji, Sandrine Lebled, Patricia Lenoble, Claudine Louesse, Ghislaine Magdelenat, Eric Mahieu, Claire Milani, Sophie Oztas, Marine Paillard, Emilie Payen, Emanuelle Petit, Murielle Ronsin, Benoit Vacherie, Alice Moussy, Corinne Cruaud, Karine Labadie, Lola Demirdjian, Simone Duprat, Emilie Téodori, Patrick Wincker, Pedro H. Oliveira, Jean-Marc Aury, Leanne Haggerty, Swati Sinha, Fergal Martin, Chiara Bortoluzzi

**Affiliations:** 1 Department of Biology, School of Sciences and Engineering, University of Crete, Vassilika Vouton, Heraklion, GR-70013, Greece; 2 Natural History Museum of Crete, School of Sciences and Engineering, University of Crete, Knossos Avenue, Heraklion, GR-71409, Greece; 3 Leibniz Institute for the Analysis of Biodiversity Change, Museum Koenig Bonn, Adenauerallee 127, Bonn, 53113, Germany; 4 Metazoa Phylogenomics Lab, Institute for Evolutionary Biology (CSIC-UPF). Passeig marítim de la Barceloneta 37-49, Barcelona, 08003, Spain; 5 Institute of Molecular Biology and Biotechnology (IMBB), Foundation for Research and Technology – Hellas (FORTH), Heraklion, GR-70013, Greece; 6 Genoscope, Institut François Jacob, CEA, CNRS, Univ Evry, Université Paris-Saclay, Evry, 91057, France; 7 Génomique Métabolique, Genoscope, Institut François Jacob, CEA, CNRS, Univ Evry, Université Paris-Saclay, Evry, 91057, France; 8 European Molecular Biology Laboratory, European Bioinformatics Institute, Wellcome Genome Campus, Hinxton, Cambridge, CB10 1SD, UK; 9 SIB Swiss Institute of Bioinformatics, Amphipôle, Quartier UNIL-Sorge, Lausanne, 1015, Switzerland

**Keywords:** Dendarus foraminosus, genome assembly, European Reference Genome Atlas, Biodiversity Genomics Europe, Earth Biogenome Project, Tenebrionidae family, Crete

## Abstract

*Dendarus foraminosus* Mulsant and Rey, 1855 is a darkling beetle in the family Tenebrionidae and one of the many *Dendarus* species endemic to the island of Crete. *Dendarus foraminosus* is a commonly found species and is widespread in the lowland and montane phrygana and maquis of central Crete. The species is classified as Least Concern (LC) by the IUCN Red List. The reference genome of *Dendarus foraminosus* will enable phylogenetic, population, and evolutionary research regarding this endemic species and its close relatives. A total of 11 contiguous chromosomal pseudomolecules (sex chromosomes included) were assembled from the genome sequence. This chromosome-level assembly encompasses 0.59 Gb, composed of 430 contigs and 415 scaffolds, with contig and scaffold N50 values of 24.4 Mb and 51.9 Mb, respectively.

## Introduction

*Dendarus foraminosus* is a darkling beetle in the family Tenebrionidae. The species is endemic to the island of Crete ( Trichas, 2008) and is mostly found in the central parts of the island, from lowland phrygana and maquis to montane shrublands. In the west- and the east-side of the island, the species is replaced by its congeneric species *D. opacus* and *D. puncticollis* respectively, while in the higher altitudes of Psiloritis and Dikti mountains by *D. politus*. All these species together form the *Dendarus* Cretan lineage ( Trichas *et al.*, 2020). According to the IUCN Red List (Red List Necca 2025, unpublished assessment), the species is not threatened by extinction, as it occurs in great abundance in many localities and various habitats in Crete.

The species larvae are saprophytophagous, participating in the decomposition of the rotting plant matter.

The reference genome of *D. foraminosus* will help in understanding the phylogenetic relationships of the species of the genus *Dendarus* in Crete, the species delimitation of the Cretan lineage, the recovery of its population structure, and the species adaptation in the Cretan environment in the face of climate change.

The generation of this reference resource was coordinated by the European Reference Genome Atlas (ERGA) initiative’s Biodiversity Genomics Europe (BGE) project, supporting ERGA’s aims of promoting transnational cooperation to promote advances in the application of genomics technologies to protect and restore biodiversity ( Mazzoni *et al.*, 2023).

## Materials & Methods

ERGA's sequencing strategy includes Oxford Nanopore Technology (ONT) and/or Pacific Biosciences (PacBio) for long-read sequencing, along with Hi-C sequencing for chromosomal architecture, Illumina Paired-End (PE) for polishing (i.e. recommended for ONT-only assemblies), and RNA sequencing for transcriptome profiling, to facilitate genome assembly and annotation.

### Sample and sampling information

**Figure 1.**
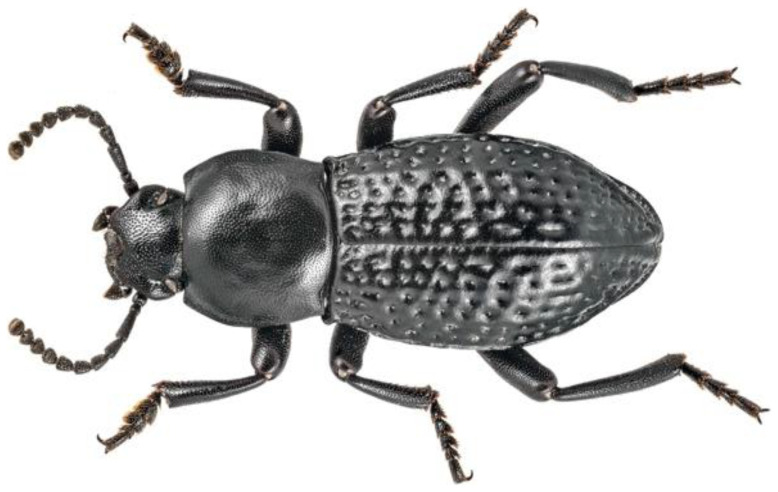
A darkling beetle ( *Dendarus foraminosus*) from a different specimen from the one sampled for this study. The specimen was photographed on the island of Crete. Photo credit: Dr. Apostolos Trichas.

On April 9th, 2022, 11 male adults of *Dendarus foraminosus* were sampled by Giannis Bolanakis ( Figure 1). The species was identified by the expert entomologist Dr. Apostolos Trichas. The specimens were hand-picked in Mount Kedros, Rethymno, Crete (Greece). Sampling was performed under the Presidential Degree 67/81 of the Greek Government. The specimens were euthanized in liquid nitrogen and were preserved at -80°C until DNA extraction.

### Vouchering information

Physical reference material for the here sequenced specimen has been deposited in the Arthropods Collections of the Natural History Museum of Crete of the University of Crete (NHMC) https://www.nhmc.uoc.gr/en/departments/arthropods/ under voucher ID NHMC.85.2.26267.

Frozen reference tissue material of abdomen, head and pronotum of *D. foraminosus* have been deposited in the Genomics and Genetic Resources Division of the NHMC https://www.nhmc.uoc.gr/ under voucher ID NHMC.85.2.26267.

### Genetic information

The estimated genome size, based on ancestral taxa, is 0.41 Gb. This is a diploid genome with a haploid number of 10 chromosomes (2n = 20). All information for this species was retrieved from Genomes on a Tree ( Challis *et al.*, 2023).

### DNA/RNA processing

DNA was extracted from a whole individual (80 mg) using a conventional CTAB extraction followed by a commercial purification using Qiagen Genomic tips (QIAGEN, MD, USA). A detailed protocol is available on protocols.io ( https://www.protocols.io/view/hmw-dna-extraction-for-long-read-sequencing-using-bp2l694yzlqe/v1).

DNA fragment size selection was performed using Short Read Eliminator (PacBio, CA, USA). Quantification was performed using a Qubit dsDNA HS Assay kit (Thermo Fisher Scientific) and integrity was assessed in a FemtoPulse system (Agilent). DNA was stored at 4 °C until usage. RNA was extracted using an RNeasy Plus Universal Kit (Qiagen) following manufacturer instructions. RNA was extracted from the whole individual (80 mg) and extracted RNA was then treated with 6U of TURBO DNase (2 U/μl) (Thermo Fisher Scientific). Quantification was performed using a Qubit RNA HS Assay and integrity was assessed in a Bioanalyzer system (Agilent). RNA was stored at -80 °C.

### Library preparation and sequencing

Long-read DNA libraries were prepared with the SMRTbell prep kit 3.0 following manufacturers' instructions and sequenced on a Revio system (PacBio). Hi-C libraries were generated from the whole individual using the Dovetail Omni-C Kit (following the Insects & marine invertebrates’ protocol v1.2) and sequenced on a NovaSeq 6000 instrument (Illumina) with 2x150 bp read length. Poly(A) RNA-Seq libraries were constructed using the Illumina Stranded mRNA Prep, Ligation Prep kit (Illumina) and sequenced on an Illumina NovaSeq 6000 instrument.

### Genome assembly methods

The genome of *Dendarus foraminosus* was assembled using the Genoscope GALOP pipeline ( https://workflowhub.eu/workflows/1200). Briefly, raw PacBio HiFi reads were assembled using Hifiasm v0.19.5-r593. Retained haplotigs were removed using purge_dups v1.2.5 with default parameters and the proposed cutoffs. The purged assembly was scaffolded using YaHS v1.2 and assembled scaffolds were then curated through manual inspection using PretextView v0.2.5 to remove false joins and incorporate sequences not automatically scaffolded into their respective locations within the chromosomal pseudomolecules. Two scaffolds with low coverage, and homology with *Tenebrio molitor* (X, Y) chromosomes, were renamed as X and Y respectively. The Telomeric repeat pattern found is TCGGG. Chromosome-scale scaffolds confirmed by Hi-C data were named in order of size. The mitochondrial genome was assembled as two circular forms using Oatk v1.0 and included in the released assembly. Summary analysis of the released assembly was performed using the ERGA-BGE Genome Report ASM Galaxy workflow ( https://doi.org/10.48546/workflowhub.workflow.1104.1).

### Genome annotation methods

A gene set was generated using the Ensembl Gene Annotation system ( Aken *et al.*, 2016), primarily by aligning publicly available short-read RNA-seq data from BioSample SAMEA112751342 to the genome. Gaps in the annotation were filled via protein-to-genome alignments of a select set of clade-specific proteins from UniProt ( UniProt Consortium, 2019), which had experimental evidence at the protein or transcript level. At each locus, data were aggregated and consolidated, prioritising models derived from RNA-seq data, resulting in a final set of gene models and associated non-redundant transcript sets. To distinguish true isoforms from fragments, the likelihood of each open reading frame (ORF) was evaluated against known metazoan proteins. Low-quality transcript models, such as those showing evidence of fragmented ORFs, were removed. In cases where RNA-seq data were fragmented or absent, homology data were prioritised, favouring longer transcripts with strong intron support from short-read data. The resulting gene models were classified into two categories: protein-coding, and long non-coding. Models that did not overlap protein-coding genes and were constructed from transcriptomic data were considered potential lncRNAs. Potential lncRNAs were further filtered to remove single-exon loci due to their unreliability. Putative miRNAs were predicted by performing a BLAST search of miRBase ( Kozomara *et al.*, 2019) against the genome, followed by RNAfold analysis ( Gruber *et al.*, 2008). Other small non-coding loci were identified by scanning the genome with Rfam ( Kalvari *et al.*, 2018) and passing the results through Infernal ( Nawrocki & Eddy, 2013).

## Results

### Genome assembly

**Figure 2.**
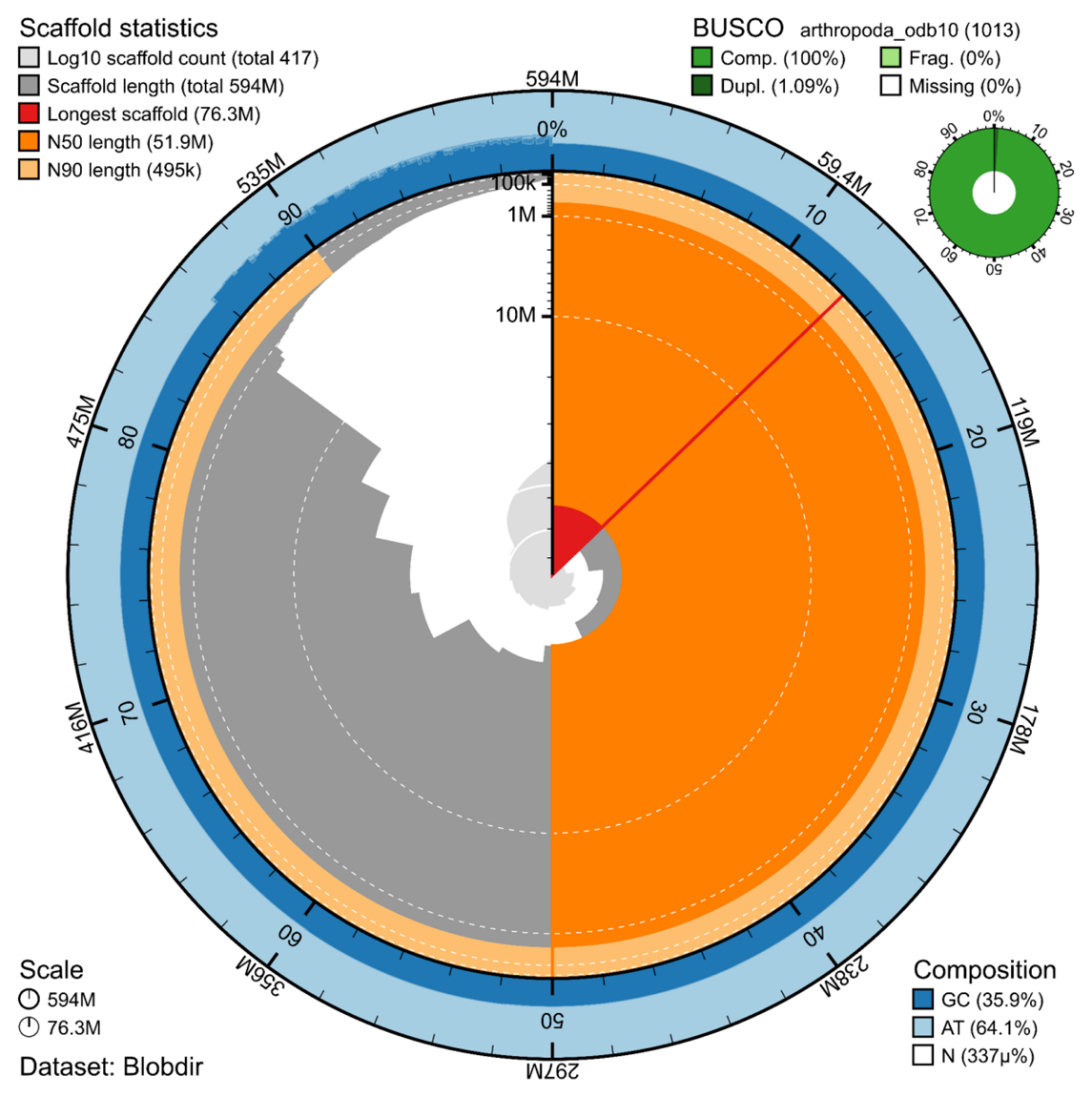
Snail plot summary of assembly statistics. The main plot is divided into 1,000 size-ordered bins around the circumference, with each bin representing 0.1% of the 593,991,149 bp assembly including the mitochondrial genome. The distribution of sequence lengths is shown in dark grey, with the plot radius scaled to the longest sequence present in the assembly (76.3 Mb, shown in red). Orange and pale-orange arcs show the scaffold N50 and N90 sequence lengths (51,873,200 bp and 494,690 bp), respectively. The pale grey spiral shows the cumulative sequence count on a log-scale, with white scale lines showing successive orders of magnitude. The blue and pale-blue area around the outside of the plot shows the distribution of GC, AT, and N percentages in the same bins as the inner plot. A summary of complete, fragmented, duplicated, and missing BUSCO genes found in the assembled genome from the Arthropoda database (odb10) is shown on the top right.

**Figure 3.**
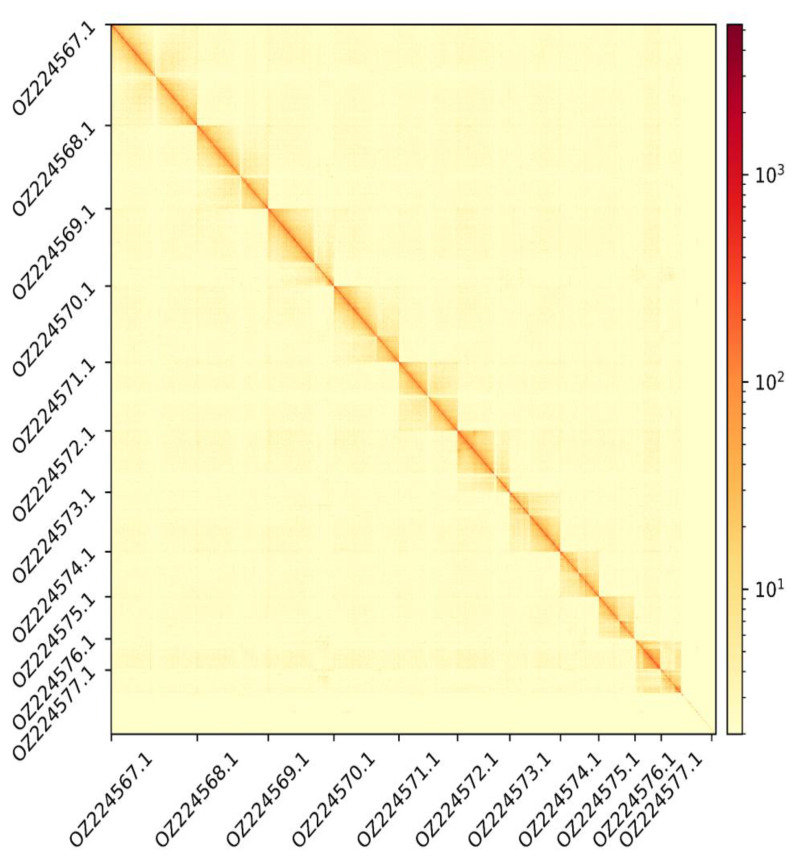
Hi-C contact map showing spatial interactions between regions of the genome. The diagonal corresponds to intra-chromosomal contacts, depicting chromosome boundaries. The frequency of contacts is shown on a logarithmic heatmap scale. Hi-C matrix bins were merged into a 100 kb bin size for plotting. On both axes the GenBank names of the 11th largest autosomes are shown.

The genome assembly has a total length of 593,991,149 bp in 415 scaffolds including two circular forms of the mitogenome ( Figure 2 and Figure 3), with a GC content of 35.9%. It features a contig N50 of 24,404,000 bp (L50 = 9) and a scaffold N50 of 51,873,200 bp (L50 = 5). There are 15 gaps, totalling 2.0 kb in cumulative size. The single-copy gene content analysis using the Arthropoda database with BUSCO ( Manni *et al.*, 2021) resulted in 100% completeness (98.9% single and 1.1% duplicated). 71.3% of reads k-mers were present in the assembly and the assembly has a base accuracy Quality Value (QV) of 63.4 calculated by Merqury ( Rhie *et al.*, 2020).

### Genome annotation

The genome annotation consists of 15,992 protein-coding genes with associated 23,634 transcripts, in addition to 2,335 non-coding genes ( Table 1). Using the longest isoform per transcript, the single-copy gene content analysis using the Arthropoda odb10 database with BUSCO resulted in 96.9% completeness. Using the OMAmer Metazoa-v2.0.0.h5 database for OMArk ( Nevers *et al.*, 2025) resulted in 93.7% completeness and 79.2% consistency ( Table 2).

**Table 1.** Statistics from assembled gene models.

	No. genes	No. transcripts	Mean gene length (bp)	No. single-exon genes	Mean exons per transcript
**mRNA**	15,992	23,634	10,478	554	5.3
**pseudogene**	0.0	0.0	0.0	0.0	0.0
**snoRNA**	22	22	116	22	1.0
**lncRNA**	1,864	1,978	4,892	3	2.2
**miRNA**	0.0	0.0	0.0	0.0	0.0
**snRNA**	71	17	136	71	1.0
**rRNA**	77	77	692	77	1.0
**scRNA**	1	1	128	1	1.0
**tRNA**	300	300	74	300	1.0
**Other ncRNA**	3	3	298	3	1.0

**Table 2.** Annotation completeness and consistency scores calculated by BUSCO run in protein mode (arthropoda_odb10) and OMArk (Metazoa-v2.0.0.h5).

	Complete	Single copy	Duplicated	Fragmented	Missing
**BUSCO**	981 (96.9%)	969 (95.7%)	12 (1.2%)	8 (0.8%)	24 (2.3%)
**OMArk**	4,465 (93.7%)	4,175 (87.6%)	290 (6.1%)	-	298 (6.2%)
	Consistent	Inconsistent	Contaminants	Unknown	
**OMArk**	12,671 (79.2%)	339 (2.1%)	0.0 (0.0%)	2,982 (18.6%)	

## Data Availability

*Dendarus foraminosus* and the related genomic study were assigned to Tree of Life ID (ToLID) 'icDenFora10' and all sample, sequence, and assembly information are available under the umbrella BioProject PRJEB77350. The sample information is available at the following BioSample accessions: SAMEA112751340, SAMEA112751341, and SAMEA112751342. The genome assembly is accessible from ENA under accession number GCA_965152765.1 and the annotated genome is available through the Ensembl website ( https://projects.ensembl.org/erga-bge/). Sequencing data produced as part of this project are available from ENA at the following accessions: ERX12116622, ERX12732899, ERX12732900, ERX12116621, and ERX12733285. ERR13362399, ERR13362013, and ERR13362014. Documentation related to the genome assembly and curation can be found in the ERGA Assembly Report (EAR) document available at https://github.com/ERGA-consortium/EARs/blob/main/Assembly_Reports/Dendarus_foraminosus/icDenFora10/. Further details and data about the project are hosted on the ERGA portal at https://www.ebi.ac.uk/biodiversity/data_portal/2712997.

## Author information

Members of the Genoscope Sequencing Team are listed here: https://zenodo.org/records/14611490.
