# Supplementary material for: ERGA-BGE genome of Dendarus foraminosus: an IUCN Least Concern darkling beetle endemic to Crete (Greece)
Source: Open Res Eur. 2026 Jan 15;5:173. Originally published 2025 Jun 30. [Version 3] doi: 10.12688/openreseurope.20489.3 (PMC12756591; doi:10.12688/openreseurope.20489.3)
Supplement: Peer review history

# Reviewer report: Reviewer response for version 3

**DOI:** 10.21956/openreseurope.24527.r68136

**Published:** 2026-01-16

**Authors:** Arun Arumugaperumal

**Affiliations:** 1 Department of Biotechnology, Rajalakshmi Engineering College, Thandalam, Chennai - 602105, Tamil Nadu, India

Figure 3 caption has been corrected. The article can be indexed.

Are sufficient details of methods and materials provided to allow replication by others?

Yes

Is the rationale for creating the dataset(s) clearly described?

Yes

Are the datasets clearly presented in a useable and accessible format?

Yes

Are the protocols appropriate and is the work technically sound?

Yes

Reviewer Expertise:

Bioinformatics; Genomics

I confirm that I have read this submission and believe that I have an appropriate level of expertise to confirm that it is of an acceptable scientific standard.

# Reviewer report: Reviewer response for version 3

**DOI:** 10.21956/openreseurope.24527.r64760

**Published:** 2026-01-16

**Authors:** Terrence Sylvester

**Affiliations:** 1 University of Memphis, Tennessee, USA

The authors have done a fantastic job of addressing my previous comments. I do not have further comments.

Are sufficient details of methods and materials provided to allow replication by others?

Partly

Is the rationale for creating the dataset(s) clearly described?

Yes

Are the datasets clearly presented in a useable and accessible format?

Yes

Are the protocols appropriate and is the work technically sound?

Yes

Reviewer Expertise:

Genomics

I confirm that I have read this submission and believe that I have an appropriate level of expertise to confirm that it is of an acceptable scientific standard.

# Reviewer report: Reviewer response for version 2

**DOI:** 10.21956/openreseurope.23682.r64761

**Published:** 2026-01-12

**Authors:** Valentina Peona

**Affiliations:** 1 Swedish Natural History Museum, Stockholm, Sweden

The authors addressed the comments fully. I do not have further comments.

Are sufficient details of methods and materials provided to allow replication by others?

Yes

Is the rationale for creating the dataset(s) clearly described?

Yes

Are the datasets clearly presented in a useable and accessible format?

Yes

Are the protocols appropriate and is the work technically sound?

Yes

Reviewer Expertise:

Evolutionary genomics, sex chromosome evolution, centromere evolution, transposable elements

I confirm that I have read this submission and believe that I have an appropriate level of expertise to confirm that it is of an acceptable scientific standard.

# Reviewer report: Reviewer response for version 2

**DOI:** 10.21956/openreseurope.23682.r66295

**Published:** 2026-01-07

**Authors:** Christopher B Cunningham

**Affiliations:** 1 University of Georgia, Atlanta, Georgia, USA

The project produced quality resources for the insect genomics community. The methods are sound and relatively well documented. The article has enough details to be reasonably reproducible with the inclusion of the pipeline documentation. The data supporting the article are publicly available.

 Abstract. Should include at least one sentence about the quality of the annotation. It would be optimal to comment on if there was anything unusual observed.  

 Keywords. These are repeated form the title and abstract so add no value. Google indexes off these too so they should reflect some broader value.

 Introduction. Background. The stated motivation for this study in the introduction is fine – part of a very large survey. However, it would be nice for the reader to understand if this is the first of its genus, is of particular interest for ecological or genetics studies, or something unusual about its biology. Helping the reader understand what this genome might help with investigation in. Most of the Intro is dedicated to a morphological description of the genus

 Methods. An empirical estimate of the genome size is needed if the author want to be confident – KAT or GenomeScope or Flow Cytometry.

 What version of OrthoDB was used? BUSCO completeness needs to be assessed with the updated OrthoDB version 12. Difference from 10 to 12 have made a big difference for several insects I work with and colleagues have reported the same.

Are sufficient details of methods and materials provided to allow replication by others?

Yes

Is the rationale for creating the dataset(s) clearly described?

Partly

Are the datasets clearly presented in a useable and accessible format?

Yes

Are the protocols appropriate and is the work technically sound?

Yes

Reviewer Expertise:

Insects, Genomics, Epigenetics, Behavior, Reproduction

I confirm that I have read this submission and believe that I have an appropriate level of expertise to confirm that it is of an acceptable scientific standard.

# Reviewer report: Reviewer response for version 2

**DOI:** 10.21956/openreseurope.23682.r66298

**Published:** 2025-12-31

**Authors:** Arun Arumugaperumal

**Affiliations:** 1 Department of Biotechnology, Rajalakshmi Engineering College, Thandalam, Chennai - 602105, Tamil Nadu, India

The data note describes the whole genome sequencing and analysis of a darkling beetle, *Dendarus foraminosus*. This is the first report of the whole genome sequence of this beetle species. The assembly reported here is of size 593.991 Mb distributed among 11 chromosomal pseudomolecules. The authors have identified the X and Y chromosomes since a male specimen had been used for sequencing. The authors have used a combination of long-read sequencing and short-read sequencing for obtaining a high quality genome assembly. The assembly contigs have been scaffolded using Hi-C data. The annotation is available as GFF3 file from Ensemble. BUSCO analysis has shown 100% completeness with respect to the arthropoda database. 

 In Figure 3 caption, it was mentioned that "On both axes the GenBank names of the 11th largest autosomes are shown". But out of the 11, two were sex chromosomes. Kindly correct it.

Are sufficient details of methods and materials provided to allow replication by others?

Yes

Is the rationale for creating the dataset(s) clearly described?

Yes

Are the datasets clearly presented in a useable and accessible format?

Yes

Are the protocols appropriate and is the work technically sound?

Yes

Reviewer Expertise:

Bioinformatics; Genomics

I confirm that I have read this submission and believe that I have an appropriate level of expertise to confirm that it is of an acceptable scientific standard.

# Reviewer report: Reviewer response for version 1

**DOI:** 10.21956/openreseurope.22173.r60054

**Published:** 2025-09-26

**Authors:** Valentina Peona

**Affiliations:** 1 Swedish Natural History Museum, Stockholm, Sweden

In this genome report, the authors present the genome assembly of the darkling beetle *Dendarus foraminosus*. The methods implemented are solid and the resulting genome assembly is of good quality.

 Minor comments

- I think it would be nice for the reader to have available a longer introduction to the species about ecological role, morphology and about why this species was chosen to be sequenced in the first place. I think the authors already introduce it but if possible it would be good to have a slightly longer introduction.
- At the end of the introduction there is a repetition of “promote”/”promoting”. I suggest to make the sentence less redundant.
- In the Genome assembly methods section, the authors mention the discovery of a telomeric repeat. I suggest to add a reference to the tool used or describe the method implemented.
- In the same section, the authors also state that the mtDNA was assembled in two circular forms. I think it would be useful to clarify if these two forms correspond to two separate haplotypes.
- Not sure but maybe the word “Degree” in the sentence “Sampling was performed under the Presidential Degree 67/81 of the Greek Government.” should be “Decree”

Are sufficient details of methods and materials provided to allow replication by others?

Yes

Is the rationale for creating the dataset(s) clearly described?

Yes

Are the datasets clearly presented in a useable and accessible format?

Yes

Are the protocols appropriate and is the work technically sound?

Yes

Reviewer Expertise:

Evolutionary genomics, sex chromosome evolution, centromere evolution, transposable elements

I confirm that I have read this submission and believe that I have an appropriate level of expertise to confirm that it is of an acceptable scientific standard, however I have significant reservations, as outlined above.

# Response

**Published:** 2025-11-10

**Authors:** Chiara Bortoluzzi

**Affiliations:** Swiss Institute of Bioinformatics, Lausanne, Vaud, Switzerland

In this revised version of the Genome Report, we have addressed all reviewer's comments as follow: 

1. As also highlighted by the other reviewer, we have now expanded on the introduction. 
2. We have rephrased the last part of the introduction to avoid repetitions. 
3.  The telomeric motif was detected using TelFinder ( https://journals.asm.org/doi/10.1128/spectrum.03928-22). The genome assembly lacks the canonical TTAGGG motif, 'TCGGG' is a putative motif. No experimental methods were used.    
4. We used Oatk to identify the mtDNA. The two forms correspond to two separate haplotypes.
5. We changed 'Degree' to 'Decree'.

# Reviewer report: Reviewer response for version 1

**DOI:** 10.21956/openreseurope.22173.r56309

**Published:** 2025-08-15

**Authors:** Terrence Sylvester

**Affiliations:** 1 University of Memphis, Tennessee, USA

In this genome report, the authors present the genome assembly of a darkling beetle, *Dendarus foraminosus*. The manuscript provides some contextual information about the species, including its distribution on the island of Crete and larval feeding patterns. However, to improve accessibility and utility—particularly for novice taxonomists—additional details on the species' morphology, life history, diagnostic features for distinguishing it from closely related species, and sexual dimorphism (e.g., how males and females can be identified) would be highly beneficial.

 In the Methods section, the authors mention that genome size was estimated based on ancestral taxa. This approach requires further clarification. Genome sizes can vary substantially even among closely related taxa, and reliance on ancestral estimates may not be sufficiently accurate for planning sequencing depth or evaluating assembly completeness. I recommend using a k-mer–based approach (e.g., using GenomeScope or similar tools) with the raw reads or including flow cytometry estimates, if available, for more accurate genome size inference.

 The manuscript states that whole bodies were used for DNA, RNA, and Hi-C library preparation. Please specify the sex of the individuals used for each of these sequencing types, as this can impact genome representation, especially with respect to sex chromosomes.

 I also suggest reporting BUSCO results before and after the removal of duplicated contigs. This would help assess whether the deduplication process led to any potential over-purging and loss of valid gene content.

 In the Results section, consider including the amount of sequencing data generated for each library type, along with the expected read depth based on the estimated genome size. These metrics are important for evaluating whether the genome assembly was sufficiently covered.

 The reported genome size of 593,991,149 bp should be expressed as 593.991 Mb for clarity and consistency with standard genome reporting practices. Similarly, assembly statistics like N50 should be reported in megabases (e.g., “13.2 Mb” rather than “13,200,000 bp”) for readability and alignment with other genome publications.

 For BUSCO, the authors should consider using a more taxonomically appropriate single-copy ortholog dataset. Using a set that is too distantly related can artificially inflate completeness scores. For this work, the Endopterygota ortholog set would be more appropriate than broader sets like Insecta or Metazoa.

 The manuscript currently lacks a clear description of assembly quality assessment methods. If such evaluations (e.g., misassembly detection, consensus accuracy, structural integrity checks) were part of the assembly pipeline, please indicate this in the Methods section. If external tools were used, list them along with their versions and relevant parameters.

 Lastly, I suggest evaluating the OZ224577.1 contig more closely to confirm that it is correctly scaffolded. In addition, the authors should label sex chromosomes (i.e., identify which scaffolds correspond to X and Y, if applicable), especially if sex-specific material was used.

 Overall, the genome of *Dendarus foraminosus* is a valuable addition to coleopteran genomics, and the assembly appears to be of good quality. Addressing the points above will greatly enhance the clarity, rigor, and reproducibility of the manuscript.

Are sufficient details of methods and materials provided to allow replication by others?

Partly

Is the rationale for creating the dataset(s) clearly described?

Yes

Are the datasets clearly presented in a useable and accessible format?

Yes

Are the protocols appropriate and is the work technically sound?

Yes

Reviewer Expertise:

Genomics

I confirm that I have read this submission and believe that I have an appropriate level of expertise to confirm that it is of an acceptable scientific standard, however I have significant reservations, as outlined above.

# Response

**Published:** 2025-11-10

**Authors:** Chiara Bortoluzzi

**Affiliations:** Swiss Institute of Bioinformatics, Lausanne, Vaud, Switzerland

In this revised version of the Genome Report, we have addressed all reviewer's comments in the following way. 

1. We have now expanded the introduction providing additional details on the species morphology, life history, diagnostic features, and sexual dimorphism. 
2. We rephrased the section about the ancestral genome size to avoid any further confusion. There are two important aspects to consider here. First, it is impractical for large collaborative initiatives, such as the European Reference Genome Atlas (ERGA), to measure genome size from sampled specimens, as these are production-based, with collaborators and collectors across Europe. Furthermore, the diversity of collector contributors, locality, budget, lack of manpower, size of specimens, and the preservation method (very often specimens are flash frozen right after field identification), makes flow cytometry impractical for all samples. The second point to consider is that estimates are obtained only for taxa and lineages with no genome size available: the estimate of genome size from GoaT is used prior to sequencing to help calculate sequencing effort (e.g. library prep reagents and number of Revio flow cells) and it is known that the value might not be exact, but it is an educated guess. The assumption that a species might have its genome size within the range described for its taxonomic group is the basis for GoaT estimates: the median of genome sizes are computed from taxa with direct measurements and propagated to the closest common ancestor, then back to fill the missing data. More information on how GoaT calculates ancestral values to propagate can be found in https://wellcomeopenresearch.org/articles/8-24/v1. K-mer genome sizes are part of our pipelines and are used routinely to assess the need for top-up sequencing during the process of producing a genome. The final assessment of a sequence-based genome size is its assembly span, which, together with K-mer inferred genome size measurements, are not the definitive amount of DNA in cells, but a reference to the size of chromosome assemblies. We have now changed the main text accordingly to clarify this aspect of the estimated genome size. 
3. We specified that Illumina and Pacbio sequencing were performed on DNA obtained from a male individual, Hi-C from a female one, and RNAseq from a second male individual. This information has now been included in the manuscript.
4. We ran BUSCO before and after the removal of duplicated contigs and results were identical in both cases. BUSCO score before purging: C:99.3%[S:98.1%,D:1.2%],F:0.1%,M:0.6%,n:3754,E:12.7%. BUSCO score after purging:  C:99.3%[S:98.1%,D:1.2%],F:0.1%,M:0.6%,n:3754,E:12.7%. Purge_dups filtered out less than 30 Mb, mainly composed of small contigs. For completeness, we reported these results in the main text. 
5. We have now included in the Results section the following sentences: “The Pacbio HiFi library generated 32.4 Gb of data, whereas Illumina libraries generated 37.5, 44.9, and 22.2 Gb of data respectively for DNAseq, Hi-C, and RNA-seq. The corresponding values of coverage are 55x, 63x, 76x, and 37x”.
6. The reported genome size and assembly statistics have been changed to megabases in the main text as requested.
7. The BUSCO completeness score when using the Endopterygota lineage was  99.2% (C:99.2%[S:98.4%,D:0.8%],F:0.5%,M:0.3%,n:2124,E:3.8%), which is not very different from the BUSCO score reported in the main text.  For completeness purposes, we reported both results in the Results section. 
8. All assembly quality assessment methods are reported in the main manuscript via the ERGA Assembly Report (EAR) document available at https://github.com/ERGA-consortium/EARs/blob/main/Assembly_Reports/Dendarus_foraminosus/icDenFora10/. This document reports all relevant information on the pre/post assembly curation process that ERGA internal reviewers will use to confirm that the assembly meets the desired quality metrics, taking into consideration peculiarities of each case. At the end of the document, tools used in the quality assessment are also reported. 
9. The internal ERGA reviewer, which is an expert in genome curation and genome assembly, did not identify any issues concerning the scaffolding of OZ224577.1. We are therefore confident that the contig was correctly scaffolded. We did not label the sex chromosomes, because, although present in the final assembly, their presence in Figure 2 would have made the Figure unreadable. That is why we decided to plot only the eleven largest autosomes. 
